# Detection of Pork in Beef Meatballs Using LC-HRMS Based Untargeted Metabolomics and Chemometrics for Halal Authentication

**DOI:** 10.3390/molecules27238325

**Published:** 2022-11-29

**Authors:** Anjar Windarsih, Florentinus Dika Octa Riswanto, Nor Kartini Abu Bakar, Nancy Dewi Yuliana, Dachriyanus, Abdul Rohman

**Affiliations:** 1 Department of Chemistry, Faculty of Science, Universiti Malaya, Kuala Lumpur 50603, Malaysia; 2 Research Center for Food Technology and Processing (PRTPP), National Research and Innovation Agency (BRIN), Yogyakarta 55861, Indonesia; 3 Division of Pharmaceutical Analysis and Medicinal Chemistry, Faculty of Pharmacy, Campus III Paingan, Universitas Sanata Dharma, Yogyakarta 55282, Indonesia; 4 Department of Food Science and Technology, IPB University, Bogor 16680, Indonesia; 5 Faculty of Pharmacy, Andalas University, Padang 25175, Indonesia; 6 Department of Pharmaceutical Chemistry, Faculty of Pharmacy, Universitas Gadjah Mada, Yogyakarta 55281, Indonesia; 7 Center of Excellence, Institute for Halal Industry and Systems (PUI-PT IHIS), Universitas Gadjah Mada, Yogyakarta 55281, Indonesia

**Keywords:** beef meatballs, chemometrics, halal authentication, pork, untargeted metabolomics

## Abstract

Adulteration of high-quality meat products using lower-priced meats, such as pork, is a crucial issue that could harm consumers. The consumption of pork is strictly forbidden in certain religions, such as Islam and Judaism. Therefore, the objective of this research was to develop untargeted metabolomics using liquid chromatography-high resolution mass spectrometry (LC-HRMS) combined with chemometrics for analysis of pork in beef meatballs for halal authentication. We investigated the use of non-targeted LC-HRMS as a method to detect such food adulteration. As a proof of concept using six technical replicates of pooled samples from beef and pork meat, we could show that metabolomics using LC-HRMS could be used for high-throughput screening of metabolites in meatballs made from beef and pork. Chemometrics of principal component analysis (PCA) was successfully used to differentiate beef meatballs and pork meatball samples. Partial least square-discriminant analysis (PLS-DA) clearly discriminated between halal and non-halal beef meatball samples with 100% accuracy. Orthogonal projection to latent structures-discriminant analysis (OPLS-DA) perfectly discriminated and classified meatballs made from beef, pork, and a mixture of beef-pork with a good level of fitness (R^2^X = 0.88, R^2^Y = 0.71) and good predictivity (Q^2^ = 0.55). Partial least square (PLS) and orthogonal PLS (OPLS) were successfully applied to predict the concentration of pork present in beef meatballs with high accuracy (R^2^ = 0.99) and high precision. Thirty-five potential metabolite markers were identified through VIP (variable important for projections) analysis. Metabolites of 1-(1*Z*-hexadecenyl)-sn-glycero-3-phosphocholine, acetyl-l-carnitine, dl-carnitine, anserine, hypoxanthine, linoleic acid, and prolylleucine had important roles for predicting pork in beef meatballs through S-line plot analysis. It can be concluded that a combination of untargeted metabolomics using LC-HRMS and chemometrics is promising to be developed as a standard analytical method for halal authentication of highly processed meat products.

## 1. Introduction

Food adulteration is becoming a global issue due to the high price of food products. Meat product is one of the food products vulnerable to adulteration because it is easy to blend meats without significantly changing the appearance and texture [1]. Beef meatballs have become the most popular meat products consumed worldwide, especially in Indonesia, Malaysia, Thailand, and Brunei Darussalam [2]. The adulteration of high-quality beef meat with lower-priced meat, such as pork meat, due to economic reasons has been carried out by unethical producers or suppliers [3]. However, pork is a non-halal meat that is prohibited from being consumed by several religions, such as Islam and Judaism. The Islamic shariah law strictly forbids the consumption of pork [4]. Apart from religion, adulteration could harm consumers who are allergic to certain meats. Adulteration of meat products is difficult to be detected because some meats have similar appearances and characteristics. Moreover, it is almost impossible to visually detect pork adulteration in highly processed food products such as beef meatballs due to the complexity of the matrices, the heterogenous composition, and the lack of morphological character [5,6]. Therefore, the development of analytical methods capable of detecting pork meat in beef meatballs with high accuracy and high efficiency is very crucial.

Numerous analytical techniques using various instrumental methods have been developed for meat products authentication, such as gas chromatography-flame ionization detector (GC-FID) [7], gas chromatography-mass spectrometry (GC-MS) [8], high-performance liquid chromatography with UV-Vis and fluorescence detector, liquid chromatography-mass spectrometry (LC-MS) [9,10], vibrational spectroscopy such as Fourier transform infrared (FTIR) spectroscopy [11], near-infrared (NIR) spectroscopy [12], Raman spectroscopy [13], DNA-based methods such as polymerase chain reaction (PCR) and real time-polymerase chain reaction (RT-PCR) as well as protein-based methods using enzyme-linked immunosorbent assay (ELISA) [14,15]. Methods based on RT-PCR have been widely used in most countries for halal authentication of meat products, such as the detection of pork in beef meatballs [3], analysis of wild boar in beef meatballs [16], and detection of dog meat in beef meatballs [17]. However, the RT-PCR technique is not without limitations. It requires rigorous sample preparation, costly reagents, and is time-consuming. In addition, it is challenging to analyze highly processed food products using RT-PCR due to the presence of food matrices and processing treatments resulting in a low amount of extractable DNA [18].

Currently, omics-based methods such as proteomics and metabolomics have been applied by most researchers in food authentication. Proteomics is one of the techniques used for meat analysis by targeting the peptide marker. Several peptide markers of pork have been reported, such as from myoglobin, carbonic anhydrase 3, beta-hemoglobin, myosin 1, and myosin 2 of pork [19]. However, proteomics is not without limitations. It requires complex sample preparation steps and is time-consuming. Moreover, it needs costly reagents, especially the use of proteolytic enzymes such as trypsin. The other approach, metabolomics, is a study for the comprehensive identification of metabolites in particular samples at a given condition. Metabolomics could be used for in-depth analysis of metabolite composition in meat samples. The difference in metabolite composition between samples provides advantages for sample differentiation. Metabolomics is closest to the phenotype, and it is known as a powerful tool for studying metabolite changes due to certain factors, including adulteration. Therefore, it can be used to provide a fingerprint pattern for each sample, which is very useful for differentiation. The application of metabolomics in food analysis has attracted great interest, including meat authentication [20]. Liquid chromatography-high resolution mass spectrometry (LC-HRMS) is known as a sophisticated analytical technique for metabolomics analysis. It offers high throughput analysis of a wide range of metabolites in samples with high specificity and sensitivity. Moreover, LC-HRMS has advantages such as simplicity in sample preparation and rapid analysis with high reproducibility [21,22]. It also provides high resolving power through the time of flight (TOF) and Orbitrap mass analyzer, which are suitable for the analysis of complex samples with high resolution and sensitivity. The high resolution of TOF has a resolving power of up to 60,000 FWHM (full width at half maximum), whereas Orbitrap has a higher resolution of up to 100,000 FWHM. However, the large data obtained from LC-HRMS measurement requires advanced statistical tools for data handling. Chemometrics is a combination of mathematical and statistical analysis for the analysis of multivariate data. Chemometrics is capable of managing and processing hundreds, even thousands, of data obtained from various instruments such as FTIR spectroscopy, GC-MS, HPLC, and LC-HRMS [23]. Chemometrics has been widely applied for metabolomics analysis, such as principal component analysis (PCA), partial least square-discriminant analysis (PLS-DA), orthogonal projection to latent structures-discriminant analysis (OPLS-DA), cluster analysis (CA), and soft independent modeling of class analogy (SIMCA). The use of chemometrics proved to be effective and exhibited some advantages in untargeted metabolomics analysis [24].

Several studies on metabolomics approaches using LC-HRMS and chemometrics for the authentication of meat and meat products have been reported. The application of metabolomics and lipidomics approach using LC-HRMS and GC-MS combined with PCA, and PLS-DA has been developed for the analysis of pork in raw beef meat. The method could detect the presence of 5% pork added to raw beef meat [25]. A study on untargeted metabolomics using LC-qTOF-MS (liquid chromatography-quadrupole time of flight-mass spectrometry) coupled with chemometrics has been used for the differentiation of goat meats obtained from pasture-fed and concentrate-fed [26]. Moreover, an untargeted metabolomics approach using LC-HRMS and chemometrics has been applied for the differentiation of chicken meats obtained from halal and non-halal slaughtering methods. Some potential metabolite markers from halal and non-halal slaughtering methods could be identified using chemometrics [27,28].

However, study on the application of the metabolomics approach for halal authentication of meat products is still limited. To our best knowledge, there is no report of the study on the detection of pork in meatballs using untargeted metabolomics and chemometrics for halal authentication. Therefore, the objective of this research was to develop an untargeted metabolomics approach using LC-Orbitrap HRMS and chemometrics for the analysis of pork in beef meatballs for halal authentication.

## 2. Results and Discussion

### 2.1. Untargeted Metabolomics Analysis Using LC-HRMS for Analysis of Meatballs

**Figure 1:**
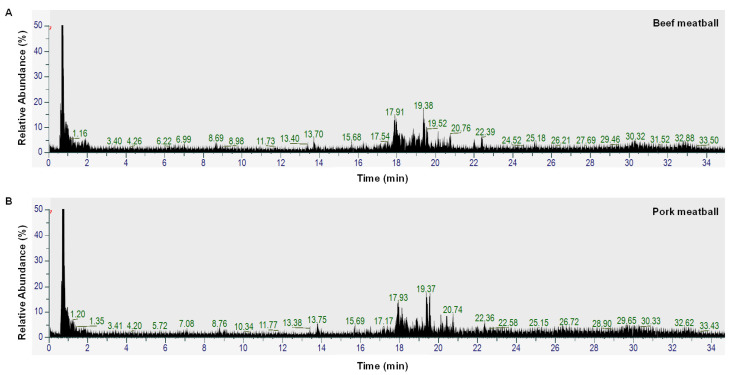
Total ion chromatogram (TIC) of a beef meatball (**A**) and a pork meatball (**B**).

The beef meatball samples have a very similar appearance and characteristics to meatballs made from pork; therefore, a visual investigation could not identify adulteration practices with pork. Analytical approaches capable of in-depth analysis of food compositions, such as metabolomics, provide advantages for the analysis of meatball adulteration based on metabolite compositions. In this study, metabolomics analysis using LC-HRMS was successfully used to identify metabolite compositions in meatball samples made from beef, pork, and mixtures of beef-pork. The total ion chromatogram (TIC) of meatballs made from beef and pork obtained from LC-HRMS measurement was very similar, as depicted in Figure 1. However, a careful visual examination could find few differences in peaks. Analysis of the metabolite composition of meatball samples extracted using methanol provided a large number of metabolites. In this study, the method could detect 559 metabolites in total from positive and negative ionization modes. Then, the data were filtered and annotated, resulting in 190 metabolites, as presented in Table S1 (Supplementary Materials), which were used as data for creating chemometrics models. Methanol was chosen as the extraction solvent because it has the capability to extract a wide range of metabolites from semi-polar to polar. It has become the most common method for metabolite extraction of plasma and tissue samples in metabolomics analysis [22].

In this study, various metabolites were found, such as amino acids, organic acids, fatty acids, nucleotides, peptides, and lipids. The threshold intensity was 1.6 × 10^5^, both in positive and negative ionization modes. The QC samples showed that the method had good stability and repeatability, demonstrated by good precision in the QC samples’ results. Amino acids and lipids were mostly found in both beef and pork meatballs. Table S2 (Supplementary Materials) demonstrates the amino acids found in meatballs made from beef and pork. Acetyl-l-carnitine, l-norleucine, and l-phenylalanine had a high peak area both in beef and pork meatballs. *N*-(tert-Butoxycarbonyl)-l-leucine and l-pyroglutamic acid was observed to have a high peak area only in beef meatballs. Meanwhile, compounds of dl-carnitine and isoleucine were found to have a high peak area only in pork meatballs. Table S3 (Supplementary Materials) shows the lipids contained in beef and pork meatballs. Compounds of 1-linoleoyl-2-hydroxy-sn-glycero-3-PC, linoleic acid, and 1-stearoylglycerol were found to be high both in beef and pork meatballs. Meanwhile, a metabolite of 1-oleoyl-2-hydroxy-sn-glycero-3-PE and PC (P-18:0/18:4 (6*Z*,9*Z*,12*Z*,15*Z*)) was found in a high area only in beef meatballs and pork meatballs, respectively. Therefore, the differentiation and classification of beef and pork meatball samples based on metabolite composition in meatballs is important for the detection of pork adulteration in beef meatballs because the composition of metabolites in adulterated meatball samples will obviously change.

A previous study reported that high-performance liquid chromatography-mass spectrometry had been successfully used to detect pork and horse meat in halal beef using targeted peptides. The method demonstrated good sensitivity, which could detect pork and horse in beef at 0.55% concentration using MRM [29]. Other peptide marker identification of pork in thermally processed meat using LC-HRMS showed valid and reliable results as well as good specificity and selectivity [18]. Detection of pork in beef meatballs has been performed using the RT-PCR technique using a Taqman probe targeting a mitochondrial D-loop. The method had good linearity and repeatability with a low limit of detection value (5 pg) [3]. However, these previous approaches are sometimes unsuitable for highly processed foods such as meatballs due to the degradation of DNA and protein. In addition, it is time-consuming and requires rigorous sample preparation. Here, we developed a potential alternative method to overcome these problems. The metabolomics approach using LC-HRMS is promising to be applied for obtaining effective and efficient halal authentication testing.

### 2.2. Analysis of Pork in Beef Meatballs Using Pattern Recognition Chemometrics

**Figure 2:**
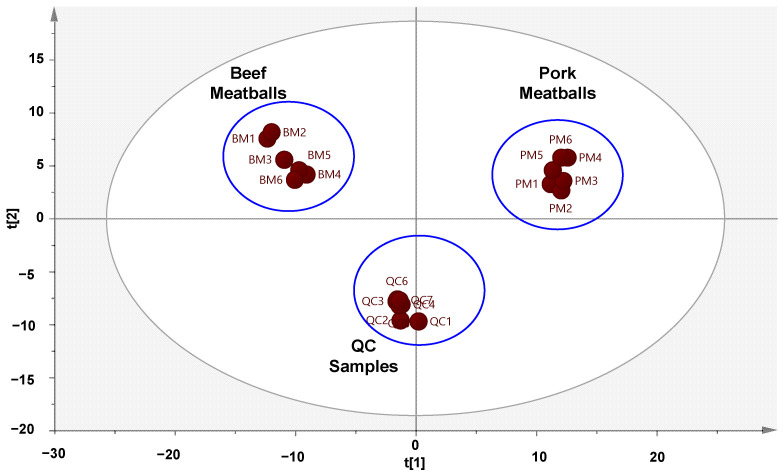
Principal component analysis (PCA) of meatballs made from beef and pork. (PC1 = 45.5%; PC2 = 24.0%).

PCA successfully differentiated meatball samples made from beef and pork, as shown in the PCA score plot (Figure 2). Beef meatballs were clearly separated from pork meatballs associated with the differences in their metabolite compositions. The PCA model was created using five principal components (PC), resulting in a good model with an R^2^ value of 0.84 and a Q^2^ value of 0.60. PCA is commonly used as the initial analysis for sample differentiation. It is categorized as an unsupervised pattern recognition which is very useful for identifying sample patterns without knowing the information or compositions of samples. However, in this study, the PCA model could not be used to clearly differentiate beef meatballs and adulterated beef meatballs with pork using several concentration levels (data not shown). Therefore, supervised pattern recognition analysis such as PLS-DA and OPLS-DA was further applied for the detection of pork in beef meatballs by discriminating meatballs made from beef, pork, and a mixture of beef-pork.

**Figure 3:**
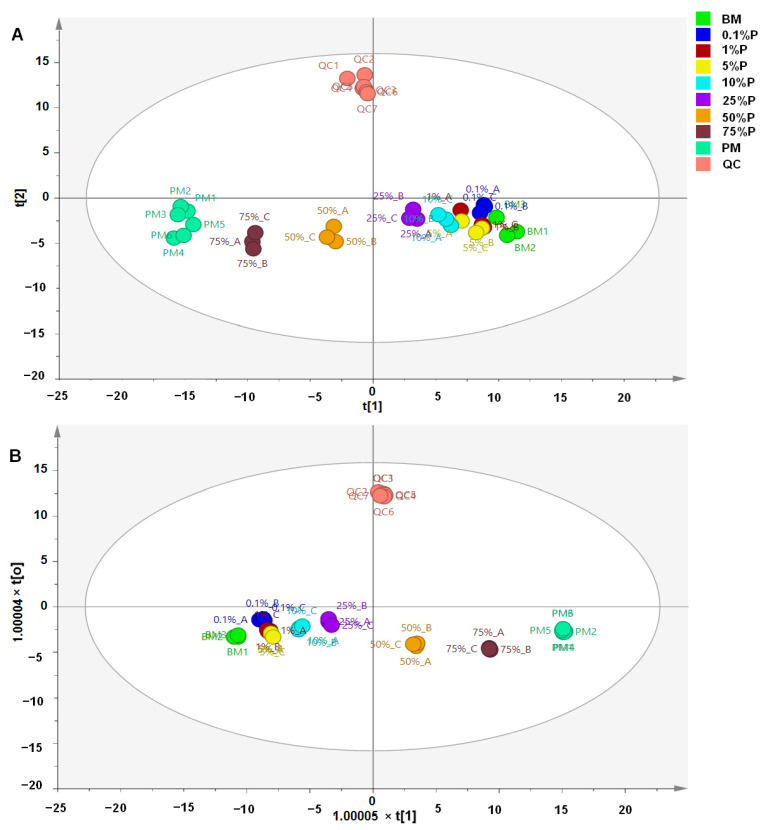
Partial least square-discriminant analysis (**A**) and orthogonal projection to latent structures-discriminant analysis (**B**) for discrimination and classification of meatballs made from beef, pork, and a mixture of beef-pork (BM = beef meatballs; PM = pork meatballs; the percentage indicates the percentage of pork added in beef meatballs).

PLS-DA using nine latent variables clearly discriminated authentic beef meatballs from adulterated beef meatballs with pork and pork meatballs, as shown in Figure 3A. All the adulterated meatball samples could be discriminated from authentic beef meatballs even in the presence of 0.1% pork mixed in a beef meatball. The PLS-DA model had R^2^X = 0.80 and R^2^Y = 0.90, indicating good fitness, which corresponded to the high accuracy of the PLS-DA model. Meanwhile, the value of Q^2^ (0.54) indicated good model predictivity. All samples were correctly classified with 100% accuracy without misclassification. PLS-DA is a combination of PLS and discriminant analysis resulting in better capability for discrimination and classification analysis [30]. It has been widely used for authentication purposes, including food origin and food adulteration. The latent variables in PLS-DA could maximize variance in PLS-DA models; therefore, a maximum separation between classes could be obtained. The PLS searches the latent variables to search the relationship between the x-matrix and y-matrix. The latent variables could maximize the variance of variables, then resulting in better classification. However, from the PLS-DA score plot, we could observe that meatballs made from 0.1%, 1%, and 5% pork appeared in a tight cluster. Even though they appeared in a tight cluster, the PLS-DA still could classify each class correctly. Apart from PLS-DA, OPLS-DA offers stronger performance for discrimination and classification among samples. It employs orthogonal variables that could enhance and maximize discrimination and classification. OPLS-DA perfectly classified authentic beef meatball samples and adulterated meatball samples with pork, as shown in the OPLS-DA score plot (Figure 3B). The OPLS-DA model was built using six predictive components and nine orthogonal x-components. The model had good accuracy (R^2^X = 0.88 and R^2^Y = 0.71) with good predictivity (Q^2^ = 0.55). The QC samples confirmed the stability of the method.

OPLS-DA was applied for the identification of metabolites, which plays important roles in sample classification through variable importance for projections (VIP) value analysis. The variables with a VIP value higher than 1 are considered to have important roles [31]. The variables obtained from VIP analysis are considered important metabolites for classification. These variables could be used as potential metabolite markers (Table 1) for differentiation of beef meatballs and pork meatballs which is important for halal authentication testing. From the 15 largest VIP values, the metabolites area of *cis*-5-tetradecenoylcarnitine, decylubiquinone, 3-methylsulfolene, (4*S*)-4-{[(9*Z*)-3-hydroxy-9-hexadecenoyl]oxy}-4-(trimethylammonio)butanoate, l-(−)-methionine, (2*E*,4*E*,16*Z*)-1-(1-Piperidinyl)-2,4,16-icosatrien-1-one, (3*β*,24*R*,24′*R*)-fucosterol epoxide, palmitoleic acid, and lysoPC (22:4 (7*Z*,10*Z*,13*Z*,16*Z*)) were significantly high in beef meatballs whereas metabolites of leu-leu, *N*-[2,5-bis (2,2,2-trifluoroethoxy)benzoyl]-*N*′-(4-methoxyphenyl)urea, Ala-Tyr, *N*-Stearoylsphingomyelin, 4-Dodecylbenzenesulfonic acid, 2,5-di-tert-butylhydroquinone had a significantly high peak area in pork meatballs.

Supervised pattern recognition, such as PLS-DA and OPLS-DA, is required to be validated to avoid overfitting the model, which leads to biased results. Validation using a permutation test employing 999 permutations has been successfully performed, confirmed the validity of the PLS-DA and OPLS-DA model shown by the intersection value of (0.0, −0.472) and (0.0, −0.533) for the PLS-DA and OPLS-DA model, respectively. The intersection of zero and below zero demonstrates model validity. Moreover, the receiver operating characteristics (ROC) test was also performed to evaluate the models’ validity. The AUC (area under the curve) of each class both in the PLS-DA and OPLS-DA model was 1, indicating correct classification in all classes, thus emphasizing model validity. It can be summarized that chemometrics of PCA, PLS-DA, and OPLS-DA could be used for authentication of beef meatballs from pork adulteration using metabolomics data obtained from LC-HRMS analysis.

### 2.3. Analysis of Pork in Beef Meatballs Using LC-HRMS and Multivariate Calibration

**Figure 4:**
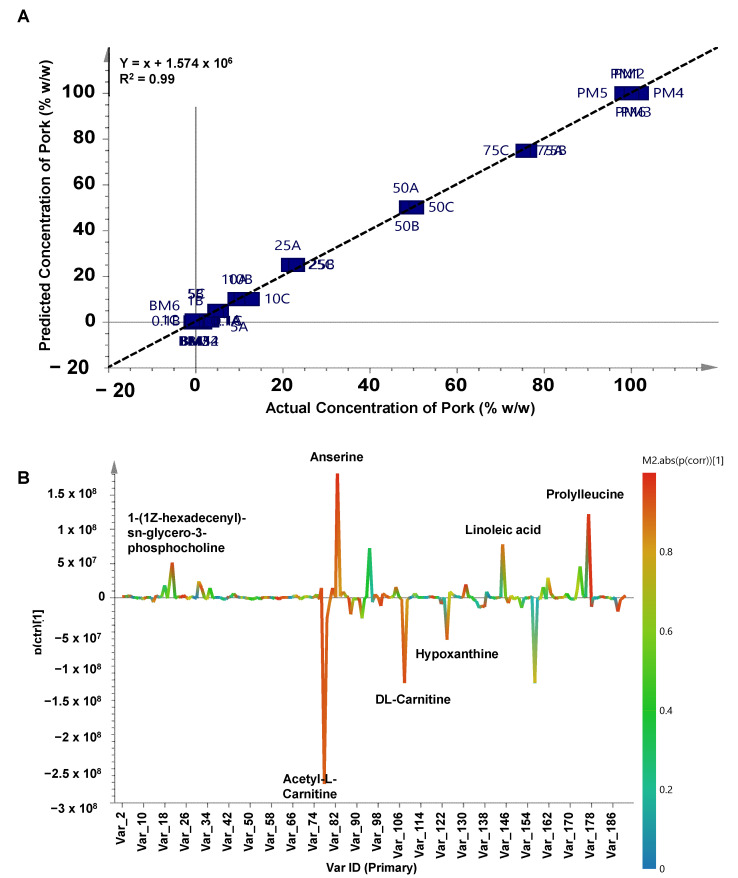
Orthogonal PLS plot (**A**) and S-line plot (**B**) to predict the concentration of pork in beef meatballs.

Multivariate calibration of PLS and OPLS was used in this study to predict the concentration of pork added to beef meatballs. PLS successfully detected and predicted the concentration of pork in beef meatballs with high accuracy (R^2^ = 0.99) using the equation of y = x + 4.597 × 10^−7^. The model error was low, as shown by the RMSEE value (1.55%) and RMSECV value (2.40%) which were associated with high precision. PLS used latent variables to search the relationship between the x-matrix (actual value) and the y-matrix (predicted value). PLS has become the most common multivariate calibration technique used to predict the concentration of target analytes using multivariate data. Apart from PLS, the OPLS technique has been introduced as the more advanced PLS technique employing orthogonal variables to search latent variables. Using one predictive component and three x-orthogonal components, OPLS was successfully used to predict the concentration of pork in beef meatballs with high accuracy and high precision. OPLS had the same R^2^ value obtained from PLS analysis, with low model error, as demonstrated by the RMSEE value (1.55%) and RMSECV value (2.30%). The OPLS plot for the correlation between the actual concentration of pork and the predicted concentration of pork is depicted in Figure 4A.

**Figure 5:**
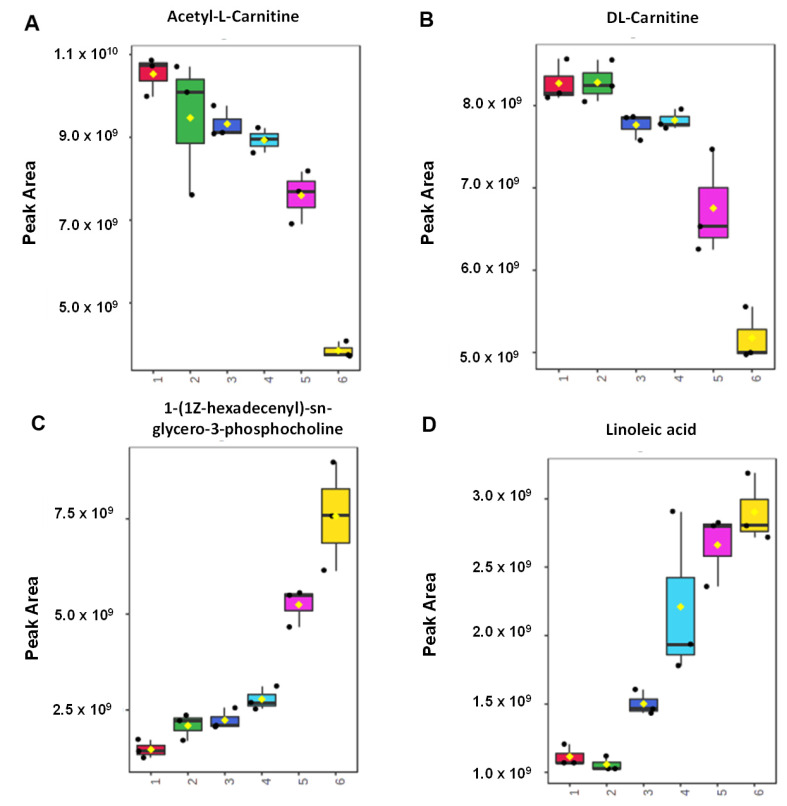
The Box-plot of (**A**) acetyl-l-carnitine, (**B**) dl-carnitine, (**C**) 1-(1*Z*-hexadecenyl)-sn-glycero-3-phosphocholine, and (**D**) linoleic acid, obtained from targeted metabolomics analysis using meatball samples containing several concentration levels of pork (1 = 100% beef, 2 = 0.1% pork, 3 = 5% pork, 4 = 25% pork, 5 = 50% pork, 6 = 100% pork).

One of the advantages of the OPLS over the PLS model is that it allows for S-line plot analysis, which is very useful for identifying the variables having important roles in the OPLS model. Variables with a high value of *p*(corr) [1], shown by the red color in the graphic, play a crucial role in the OPLS model [32]. Observed using an S-line plot in Figure 4B, variables of 1-(1*Z*-hexadecenyl)-sn-glycero-3-phosphocholine, acetyl-l-carnitine, anserine, dl-carnitine, linoleic acid, hypoxanthine, and prolylleucine identified as important metabolites responsible in OPLS model for predicting the concentration of pork in beef meatballs. Some metabolites obtained from the S-line plot differ from VIP analysis because they were obtained from a different approach and have a different role. Metabolites in Table 1 are associated with discriminating metabolites for sample classification, whereas metabolites from the S-line plot indicated their role in predicting the concentration of pork contained in beef meatballs. The targeted metabolomics analysis of important metabolites from the S-line plot has been carried out in this study for samples prepared using several concentration levels of pork (0%, 0.1%, 10%, 25%, 50%, and 100%) and the result showed that compounds of acetyl-l-carnitine and dl-carnitine had high peak areas in beef meatballs (Figure 5) and significantly decreased as the concentration of pork increased. Meanwhile, metabolites of 1-(1*Z*-hexadecenyl)-sn-glycero-3-phosphocholine and linoleic acid were found to be significantly increased as the level of pork in beef meatballs increased (Figure 5). Therefore, multivariate calibration of PLS and OPLS has the capability to process the metabolites data obtained from untargeted LC-HRMS measurement for detection and prediction of the amount of pork present in beef meatballs. It is very useful to be developed and used as an analytical method for halal authentication of meat-based food products.

## 3. Materials and Methods

### 3.1. Materials

Methanol for LC-MS, water for LC-MS, acetonitrile for LC-MS, methanol for HPLC, and formic acid was obtained from Merck (Darmstadt, Germany). Pierce positive calibration solution (Thermo Scientific, Rockford, IL, USA) and Pierce negative calibration solution (Thermo Scientific, Rockford, IL, USA) were obtained from Thermo Fischer (Thermo Scientific, Rockford, IL, USA).

### 3.2. Sample Collection and Preparation

Beef meat was purchased from five halal slaughtering houses in Yogyakarta and Central Java, Indonesia. The pork meat was purchased from three pig slaughterhouses in Yogyakarta, Indonesia. The beef and pork samples were from three individuals from each slaughterhouse and obtained from a loin cut. The weight of selected beef and pork was 300–350 kg and 80–85 kg, respectively. The meats were stored at −20 °C prior to being used for analysis. Preparation of the meatball samples was according to Kuswandi et al. [33] and Raharjo et al. [34] with slight modifications. The samples were defrosted/thawed in a refrigerator (5 °C) for 6 h. The minced meat of beef from five slaughterhouses was pooled into one place and homogenized, whereas the minced pork from three slaughterhouses was also pooled into one place and homogenized. The meatball samples were made from each pooled beef and pork sample. The composition of the meatball samples was 90% meat and 10% other ingredients, consisting of tapioca flour and salt. The series of adulterated beef meatballs with pork was prepared by mixing pork and beef at several concentration levels. The concentration of pork added was 0%, 0.1%, 0.5%, 1%, 10%, 25%, 50%, 75%, and 100% (*w*/*w*). The total weight of each meatball was 5 g. Subsequently, meatball samples were cooked by boiling for 30 min at 100 °C.

### 3.3. Metabolite Extraction

Meatballs were ground using a mortar and stamper, then placed into a 50 mL centrifuge tube. Extraction was performed by adding 25 mL of HPLC-grade methanol. The mixture was vortexed for 2 min, then ultrasonicated at room temperature for 30 min. After the sonication process, samples were stored at −20 °C for 1 h to precipitate proteins. Then, 1 mL of the supernatant was taken and centrifuged at 5000 rpm for 5 min to separate the pellet and the supernatant. The supernatant was filtered through a PTFE filter at 0.22 µm and placed into a 2 mL HPLC vial. The samples were ready to be used for LC-HRMS analysis. Quality control (QC) samples were prepared by collecting 50 µL supernatant from each sample series (*n* = 27). QC sample was used to evaluate the stability and repeatability of the method. The sample was injected every 6 injections.

### 3.4. Metabolomics Analysis Using Liquid Chromatography-High Resolution Mass Spectrometry

Metabolomics analysis was carried out according to the method by Windarsih et al. [35] with slight modifications. Analysis was performed using a binary pump UHPLC Vanquish (Thermo Scientific, Rockford, IL, USA) coupled with high-resolution mass spectrometry Q-Exactive Orbitrap (Thermo Scientific, Rockford, IL, USA). An analytical column of Accucore C-18 (10 mm × 2.6 mm ID × 2.1 µm) was used for metabolite separation. The column temperature was set at 40 °C during analysis. The mobile phase used for analysis was water containing 0.1% formic acid (A) and methanol containing 0.1% formic acid (B) employing a gradient technique with a flow rate of 0.30 mL/min, and the total running time was 35 min. The mobile phase was set at 95% A at the initial condition, then continued to 90% B at 16 min. The condition at 90% B was maintained for 10 min before going back to the initial condition. Each sample was injected at a volume of 10 µL. The QC sample was injected every 6 sample injections. For mass spectrometric conditions, the sheath gas flow rate was set at 32 arbitrary units (AU) with auxiliary and sweep gas flow rates of 8 and 4 AU, respectively. The scanning was performed both in MS1 and MS2, with the resolution used for MS1 being 70,000 while the resolution for MS2 was 17,500. The collision energy (NCE) used was 10 eV. Analysis was performed both in positive ionization mode and negative ionization mode simultaneously. Scanning of the metabolites was performed at the 66.7–1000 *m*/*z* range. The mass calibration was performed routinely using positive and negative ESI calibration solutions to maintain the performance and accuracy of the mass analyzer.

### 3.5. Metabolite Analysis

Metabolite analysis was carried out using Compound Discoverer software (Thermo Scientific, Rockford, IL, USA) to identify the metabolite compositions. The total ion chromatograms (TIC) of both samples and blank imported from X-Calibur were analyzed using Compound Discoverer for background drift correction, alignment of retention time, peak extraction, peak integration, feature detection, and database matching. First, background spectra were used for correction analysis by subsequent subtraction of blank. Then, the compounds were searched against the databases of MzCloud and Chemspider for peak extraction. The compounds with error mass between −5 ppm and 5 ppm compared to the MzCloud library were selected. Subsequently, the compounds with a retention time tolerance of 0.2 min compared to MzCloud were retained. Then, only compounds with a full match with MzCloud with MzCloud best match >70 and a full match with Chemspider were selected. The peak Intensities were normalized according to the total spectra Intensity.

### 3.6. Chemometrics Analysis

The peak area of metabolite compositions was used as the variable for chemometrics analysis. SIMCA 14.0 software (Umetrics, Umea, Sweden) was used for chemometrics analysis. Chemometrics of either pattern recognition or multivariate calibration was applied in this study. The number of metabolites used for chemometrics analysis was 190 metabolites, and the input variables were the peak area value from each metabolite. Prior to chemometrics analysis, the data were normalized using sum normalization and scaled using the UV scaling method. No data transformation was performed. Principal component analysis (PCA), partial least square-discriminant analysis (PLS-DA), and orthogonal projection to latent structures-discriminant analysis (OPLS-DA) were used for pattern recognition. Meanwhile, partial least square (PLS) and orthogonal PLS (OPLS) were applied for multivariate calibration. The PCA was observed using the PCA score plot, R^2^, and Q^2^ values. PLS-DA and OPLS-DA were used for sample discrimination and classification. Models were evaluated using the score plot, R^2^X, R^2^Y, Q^2^, permutation test, and receiver operating characteristics (ROC) test. Cross-validation using the leave-one-out technique was used in this study. The data were scaled using the UV scaling technique prior to chemometrics analysis. The identification of important variables for sample differentiation was performed using variable importance for projections (VIP) values. In addition, PLS and OPLS were intended to predict the concentration of pork added to beef meatballs. The performance of the PLS model was observed using a PLS plot, RMSEE (root mean square error of estimation), and RMSECV (root mean square error of cross-validation). An S-line plot was used to identify the important variables responsible for predicting pork concentration in the OPLS model.

## 4. Conclusions

Adulteration of beef meatballs with pork is a fraudulent practice that affects the halal status and quality of food products. The untargeted metabolomics approach using liquid chromatography-high resolution mass spectrometry employing Orbitrap mass analyzer could be used for the identification of metabolites in meatball samples made from beef, pork, and a mixture of beef-pork. A combination with chemometrics of PCA could be used for the detection of adulteration in beef meatballs by differentiating pure beef meatballs and beef meatballs containing pork. PLS-DA and OPLS-DA successfully detected and clearly classified authentic beef meatballs and adulterated beef meatballs with pork. A number of metabolites (35 metabolites) were found as potential metabolite markers for meatballs differentiation identified using VIP analysis. In addition, multivariate calibration of PLS and OPLS has been successfully used to detect and predict the concentration of pork in beef meatball samples. The lowest concentration of pork that could be detected using the developed methods in this study was 0.1% (*w*/*w*). Metabolites of 1-(1*Z*-hexadecenyl)-sn-glycero-3-phosphocholine, acetyl-l-carnitine, dl-carnitine, anserine, hypoxanthine, linoleic acid, and prolylleucine were identified to have an important role in predicting pork contained in beef meatballs. It can be concluded that a combination of untargeted metabolomics using LC-HRMS in combination with advanced chemometrics could be used as a rapid, effective, and efficient analytical method for the analysis of pork in meat products. It is promising to be developed as a standard analytical method for halal authentication of highly processed meat products. Further analysis of larger samples is required to emphasize the reproducibility of the analytical method.

## Figures and Tables

**Table 1:** Potential metabolite markers for differentiation of meatballs made from beef, pork, and a mixture of beef-pork obtained from VIP analysis.

No.	Metabolites	VIP Value	Ionization Mode
1.	*Cis*-5-Tetradecenoylcarnitine	1.67	+
2.	Leu-leu	1.56	+
3.	Decylubiquinone	1.55	−
4.	3-Methylsulfolene	1.51	+
5.	(4*S*)-4-{[(9*Z*)-3-Hydroxy-9-hexadecenoyl] oxy}-4-(trimethylammonio) butanoate	1.49	+
6.	l-(−)-Methionine	1.43	+
7.	*N*-[2,5-bis (2,2,2-trifluoroethoxy) benzoyl]-*N*′-(4-methoxyphenyl) urea	1.42	+
8.	Ala-Tyr	1.35	+
9.	(2*E*,4*E*,16*Z*)-1-(1-Piperidinyl)-2,4,16-icosatrien-1-one	1.34	+
10.	(3beta,24*R*,24′*R*)-fucosterol epoxide	1.32	+
11.	Palmitoleic acid	1.31	−
12.	*N*-Stearoylsphingomyelin	1.30	+
13.	4-Dodecylbenzenesulfonic acid	1.22	−
14.	2,5-di-tert-Butylhydroquinone	1.22	−
15.	LysoPC (22:4(7*Z*,10*Z*,13*Z*,16*Z*))	1.21	+
16.	1-[(9*Z*)-octadecenyl]-2-hexadecanoyl-sn-glycero-3-phosphocholine	1.21	+
17.	*Trans*-10-Heptadecenoic acid	1.21	−
18.	Threonylphenylalanine	1.20	+
19.	Nicotinic acid	1.19	+
20.	Succinic anhydride	1.19	+
21.	Inosine-5′-monophosphate (IMP)	1.17	+
22.	(15*Z*)-9,12,13-Trihydroxy-15-octadecenoic acid	1.16	−
23.	1,2-Dilauroyl-sn-glycero-3-PE	1.16	+
24.	Betaine	1.15	+
25.	(+/−) 9-HpODE	1.15	−
26.	3-Hydroxy-3-[(3-methylbutanoyl) oxy]-4-(trimethylammonio) butanoate	1.15	+
27.	α-Eleostearic acid	1.15	+
28.	Acetyl-l-carnitine	1.14	+
29.	*N*-tert-Butyloxycarbonyl-deacetyl-leupeptin	1.14	+
30.	PC (*o*-18:1 (9*Z*)/18:2 (9*Z*,12*Z*))	1.14	+
31.	Docosapentaenoic acid	1.14	−
32.	Arabinosylhypoxanthine	1.13	−
33.	Monoolein	1.12	+
34.	Prolylleucine	1.11	+
35.	Propionylcarnitine	1.10	+

## Data Availability

The data presented in this study are available on request from the corresponding author.

## Supplementary Materials

The following supporting information can be downloaded at: https://www.mdpi.com/article/10.3390/molecules27238325/s1, Table S1. The metabolites of meatballs made from beef, pork, mixtures of beef-pork analyzed using LC-HRMS method; Table S2. The amino acid compounds contained in beef and pork meatballs; Table S3. The fatty acid and lipid compounds contained in beef and pork meatballs.

## Sample Availability

Samples of the compounds are available from the authors.
